# Comparison of the metal accumulation capacity between the acanthocephalan *Pomphorhynchus laevis* and larval nematodes of the genus *Eustrongylides* sp. infecting barbel (*Barbus barbus*)

**DOI:** 10.1186/1756-3305-6-21

**Published:** 2013-01-18

**Authors:** Milen Nachev, Gerhard Schertzinger, Bernd Sures

**Affiliations:** 1 Aquatische Ökologie (Aquatic ecology) and Zentrum für Wasser- und Umweltforschung (ZWU), Universität Duisburg-Essen, Universitätsstraße 5, D-45141, Essen, Germany

**Keywords:** *Pomphorhynchus laevis*, *Eustrongylides* sp., Heavy metals, Pollution, Bioindication

## Abstract

**Background:**

Metal uptake and accumulation in fish parasites largely depends on the parasite group with acanthocephalans showing the highest accumulation rates. Additionally, developmental stage (larvae or adult) as well as parasite location in the host are suggested to be decisive factors for metal bioconcentration in parasites. By using barbel (*Barbus barbus*) simultaneously infected with nematode larvae in the body cavity and adult acanthocephalans in the intestine, the relative importance of all of these factors was compared in the same host.

**Methods:**

Eleven elements Arsenic (As), Cadmium (Cd), Cobalt (Co), Copper (Cu), Iron (Fe), Manganese (Mn), Lead (Pb), Selenium (Se), Tin (Sn), Vanadium (V) and Zinc (Zn) were analyzed in barbel tissues (muscle, intestine, liver) as well as in their acanthocephalan parasites *Pomphorhynchus laevis* and the larval nematode *Eustrongylides* sp. (L4) using inductively coupled plasma mass spectrometry (ICP-MS).

**Results:**

Nine elements were detected in significantly higher levels in the parasites compared to host tissues. The element composition among parasites was found to be strongly dependent on parasite taxa/developmental stage and localization within the host. Intestinal acanthocephalans accumulated mainly toxic elements (As, Cd, Pb), whereas the intraperitoneal nematodes bioconcentrated essential elements (Co, Cu, Fe, Se, Zn).

**Conclusion:**

Our results suggest that in addition to acanthocephalans, nematodes such as *Eustrongylides* sp. can also be applied as bioindicators for metal pollution. Using both parasite taxa simultaneously levels of a wide variety of elements (essential and non essential) can easily be obtained. Therefore this host-parasite system can be suggested as an appropriate tool for future metal monitoring studies, if double infected fish hosts are available.

## Background

Different endohelminths of fish were suggested as sentinel organisms to detect metal pollution in aquatic habitats [1]. Most of the available papers emphasize adult intestinal parasites such as acanthocephalans (e.g. *Pomphorhynchus laevis, Acanthocephalus lucii*), which have a remarkable capacity to accumulate heavy metals [1,2] at concentrations many hundred to a thousand times higher than their host tissues and the aqueous environment [3,4]. With the use of these parasites even low levels of environmental pollution can be detected [5], which is often impossible using conventional analytical methods and/or free living sentinels such as mussels [6]. Their excellent accumulation capacity was found to be related to the acanthocephalans’ anatomy, metabolism/physiology and localization in the host [4,7].

Metal accumulation in parasitic nematodes appears to be more variable than in acanthocephalans with usually only slightly higher concentrations of a few elements in adult *Philometra cyprinirutili*, *P. ovata*, *Anisakis simplex* and *Anguillicola crassus* in comparison to their fish host tissues [8-12]. Less information is available on metal accumulation rates in larval fish nematodes. It could be expected, however, that each developmental stage shows different metal accumulation profiles as e.g. larval stages have a different metabolic activity. Moreover, nematode larvae are often encapsulated in host tissues, which could restrict uptake of nutrients and pollutants. The microhabitat preference (localization in the fish host) could also affect metal accumulation, due to an organ-specific availability of metals. As suggested by Sures & Siddall [7] the major route of metal uptake in the fish occurs in the gills. Subsequently, metals are transported by the circulatory system into the fish liver where they are bound in bile complexes and excreted into the intestine. Here, they become available for the parasites located in the intestine. Accordingly, intestinal parasites should have a better access to metals than parasites dwelling in the body cavity. This assumption was tested in laboratory exposure experiments with the acanthocephalan *P. laevis*[13]. Individuals which penetrated the intestinal wall and which were found in the cavity of fish accumulated significantly less lead than the acanthocephalans in the intestinal lumen.

The aim of the present study was to evaluate metal accumulation of different fish helminths inhabiting different microhabitats within the same host. As a suitable fish host barbel, *Barbus barbus*, infected with larval nematodes of the genus *Eustrongylides* sp. and the adult acanthocephalan *P. laevis* were investigated. Barbel serves as second intermediate or paratenic host for the nematodes that were located in the anterior part of the body cavity, mainly on the serosa of the intestine and in the liver tissue. In most cases, the nematodes were surrounded by a capsule, forming a spiral granuloma, as described by Mihalca et al. [14]. Simultaneously, free moving nematodes were found, which appeared to cause massive histological damage such as penetrations of the cavity wall and disruptions of inner organs. The adult acanthocephalans were located in the lumen of the small intestine and were therefore directly exposed to bile fluids and the food acquired by the fish host. This system of one host infected with two different parasites is well suited to comparatively assess the relevance of the parasite’s taxonomic position, developmental stage and the location within its host for metal accumulation.

## Methods

### Fish samples

Fish infected simultaneously with *P. laevis* and larval nematodes of the genus *Eustrongylides* sp. were identified during a four years sampling of barbel from the Bulgarian part of the river Danube (for details see [15]). Due to lower prevalence of the nematodes (between 17 and 24%) compared to 98 – 100% prevalence for *P. laevis*, 16 double infected barbel were selected. From these fish, samples of muscle (anterior part of body), intestine (medial section of intestinal tract) and liver as well as the parasites were taken and frozen at – 20°C until further processing for metal analysis.

### Metal analysis

The preparation of samples for metal analysis was performed according to the procedure described earlier [16,17]. After thawing, 150 to 340 mg (wet weight) of the homogenized fish tissues and up to 110 mg of parasites was weighed into reaction vessels. The larval nematodes were previously extracted from the cysts, if they were encapsulated. A mixture of 1.3 ml nitric acid (65% HNO_3_, suprapure) and 2.5 ml hydrogen peroxide (30% H_2_O_2_, suprapure) was added and the vessels were heated for 90 min at about 170 °C using the microwave digestion system MARS 5 (CEM GmbH, Kamp-Lintfort, Germany). After digestion the clear sample solution was brought to 5 ml volume with deionised water (MiliPore) in a volumetric glass flask. Subsequently, the concentrations of As, Cd, Co, Cu, Fe, Mn, Pb, Sn, Se, V, Zn were analyzed using inductively coupled plasma mass spectrometry (ICP-MS, Perkin Elmer, Elan 5000; details on instrumental settings, calibration and sample measurements are given in [17]).

For validation of the analytical procedure a standard reference material (DORM-3, National Research Council, Canada) was digested and analyzed in the same way as the fish samples and the certified values of 7 elements were compared (Table 1). Detection limits (DL) for all elements were determined following standard procedures.

**Table 1:** Trace metal concentrations in certified reference material (DORM-3) as well as accuracy and detection limits determined by ICP-MS analyses

**Element**	**DORM-3 values**	**DORM-3 analyzed**	**Accuracy**	**Detection limit**	**Detection limit (mg/kg)**
	**± SD (mg/kg)**	**± SD (mg/kg)**	**(%)**	**(μg/L)**	**for 200 mg (FW) sample**
As	6.88 ± 0.30	6.30 ± 0.40	92%	0.008	0.001
Cd	0.290 ± 0.020	0.27 ± 0.02	94%	0.01	0.001
Co	n.c.	-	-	0.009	0.001
Cu	15.5 ± 0.63	16.35 ± 0.93	105%	0.19	0.078
Fe	347 ± 20	346.95 ± 28.24	100%	2.76	0.354
Mn	n.c.	-	-	0.1	0.251
Pb	0.395 ± 0.050	0.417 ± 0.043	106%	0.26	0.003
Se	n.c	-	-	0.14	0.008
Sn	0.066 ± 0.012	0.0067 ± 0.010	102%	0.01	0.005
V	n.c.	-	-	0.01	0.002
Zn	51.3 ± 3.1	44.4 ± 3.2	87%	2.77	0.161

FW: fresh weight.

### Data analyses and statistical treatment

In order to express the accumulation capacity of parasites the ratios C_[parasite]_/C_[host tissue]_ (Bioconcentration factors - BCF) were calculated according to Sures et al. [18]. Additionally, the concentration ratio between *Eustrongylides* sp. and *P. laevis* was determined. In order to test for significant differences between tissues and parasites Friedman’s ANOVA and Wilcoxon matched pair tests were applied. Spearman rank correlation was calculated to check for possible relationship/interactions between element concentrations in different organs and parasites. All statistic methods were performed with STATISTICA 9.0.

## Results

### Analytical procedure

The accuracy of certified elements in DORM3 as well as the detection limits of each element are listed in Table 1. The accuracy varied between 87% and 106%, which can be considered a reliable analysis. The detection limits calculated for essential metals (e.g. Cu, Fe, Mn and Zn) were higher than those for non-essential ones (see Table 1) due to their higher natural occurrence.

### Element concentrations in the host-parasite system

**Figure 1:**
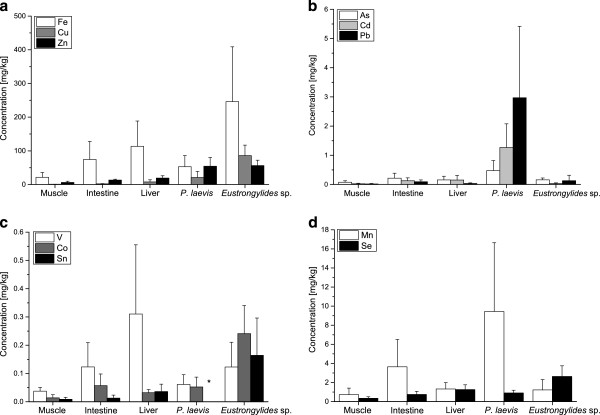
Mean (±S.D.) element concentrations (a–d) in organs of barbels and in its parasites *Pomphorhynchus laevis *and *Eustrongylides *sp. *Concentrations are not displayed, as they were below the detection limit

The distribution of elements in the host-parasite system is displayed in Figure 1. Nine of eleven elements were found in significantly higher concentrations in the parasites (either in *P. laevis* or in *Eustrongylides* sp.) compared to their host tissues (Friedman’s ANOVA, p < 0.05). The concentrations of the toxic elements (e.g. As, Cd, Pb) in fish tissues were below 0.5 mg/kg (Figure 1b), whereas essential elements in some cases exceeded concentrations of 50 mg/kg (e.g. Cu). The acanthocephalans showed significantly higher levels of As, Cd, Cu, Mn, Pb and Zn compared to all host tissues (Wilcoxon test, p ≤ 0.01; Table 2) with bioconcentration factors of up to 340 (Table 3). Correlation analyses between element concentrations in host tissues and acanthocephalan showed some significant associations for the elements Co, Mn, Pb, Se and V (Table 4). Interestingly, positive correlations were found between the parasites and the intestine or liver (e.g. Co, Mn, Pb and V), respectively. The only negative relationship was obtained for Se concentrations between *P. laevis* and host muscle (r = − 0.51; p < 0.05).

**Table 2:** **Differences between element concentrations in barbel organs and parasites, and between *Pomphorhynchus laevis *and *Eustrongylides *sp**

	***P. laevis***			***Eustrongylides *sp.**			***P.laevis *vs *Eustrongylides *sp.**
**Element**	***P.l. *↔ M**	***P.l. *↔ I**	***P.l. *↔ L**	**E ↔ M**	**E ↔ I**	**E ↔ L**	**E ↔ P.l.**
As	P.l. **	P.l. **	P.l. **	E*	n.s.	n.s.	P.l.**
Cd	P.l. **	P.l. **	P.l. **	n.s.	I**	L**	P.l.*
Co	P.l.**	n.s.	P.l.*	E**	E**	E**	E**
Cu	P.l. **	P.l. **	P.l. **	E**	E**	E**	E**
Fe	P.l.**	n.s.	L**	E**	E**	E**	E**
Mn	P.l. **	P.l. **	P.l. **	n.s.	I**	n.s.	P.l.**
Pb	P.l. **	P.l. **	P.l. **	E**	n.s.	E**	P.l.**
Se	P.l.**	n.s.	n.s.	E**	E**	E**	E**
Sn	M^1^	I^1^	L^1^	E*	n.s.	E*	E^**^
V	P.l.**	I**	L**	E**	n.s.	L**	E**
Zn	P.l. **	P.l. **	P.l. **	E**	E**	E**	n.s.

M: muscle; I: intestine; L: liver; P.l.: *Pomphorhynchus laevis*; E: *Eustrongylides* sp.

*: significant at p ≤ 0.05 (Wilcoxon matched pair test).

**: significant at p ≤ 0.01 (Wilcoxon matched pair test).

^1^ : not statistically tested as concentrations in *Pomphorhynchus laevis* were below the detection limit.

n.s.: not significantly different (Wilcoxon matched pair test).

In case of significant difference, the site for higher concentration is given in each case.

**Table 3:** **Bioconcentration factors C**_**[parasite] **_**/ C**_**[barbel tissue]**_**for *Pomphorhynchus laevis *and *Eustrongylides *sp. calculated with respect to different host tissues and ratios C**_**[*Eustrongylides*sp.] **_**/ C**_**[*P. laevis*]**_

	***P. laevis *C**_**[*P.laevis*] **_**/ C**_**[Organ]**_ **± SD**			***Eustrongylides *sp. C**_**[*Eustrongylides*sp.] **_**/ C**_**[Organ]**_ **± SD**			**C**_**[*Eustrongylides*sp.] **_**/ C**_**[*P. laevis*]**_ **± SD**
	**Muscle**	**Intestine**	**Liver**	**Muscle**	**Intestine**	**Liver**	
As	7.6 (± 7.4)	3.3 (± 3.6)	4.5 (± 4.9)	3.0 (± 2.1)	1.0 (± 0.6)	2.0 (± 2.2)	0.6 (± 0.6)
Cd	90.3 (± 121.6)	15. 6 (± 13.3)	14.1 (± 10.7)	1.3 (± 0.8)	0.3 (± 0.2)	0.3 (± 0.2)	0.04 (± 0.04)
Co	5.3 (± 3.8)	1.2 (± 0.7)	1.6 (± 0.8)	24.1 (± 12.1)	6.6 (± 5.2)	8.3 (± 2.9)	5.8 (± 3.8)
Cu	26.0 (± 26.5)	11.0 (± 10.2)	4.5 (± 6.5)	122.9 (± 71.0)	48.3 (± 22.6)	20.7 (± 17.8)	6.9 (± 5.2)
Fe	3.3 (± 2.8)	0.8 (± 0.4)	0.7 (± 0.6)	14.3 (± 7.7)	3.8 (± 2.1)	2.9 (± 1.7)	4.8 (± 2.0)
Mn	20.6 (± 18.5)	3.9 (± 4.5)	7.1 (± 4.1)	2.2 (± 1.3)	0.4 (± 0.3)	1.0 (± 0.7)	0.2 (± 0.1)
Pb	337 (± 401)	36.8 (± 27.6)	142 (± 133)	9.4 (± 12.5)	1.4 (± 1.8)	4.5 (± 4.6)	0.04 (± 0.03)
Se	2.5 (± 1.5)	1.2 (± 0.5)	0.8 (± 0.4)	7.1 (± 4.9)	3.4 (± 1.9)	2.1 (± 1.0)	2.9 (± 1.2)
Sn	n.d.	n.d.	n.d.	54.5 (± 93.4)	22.1 (± 23.3)	9.1 (± 8.5)	n.d.
V	2.0 (± 1.7)	0.6 (± 0.3)	0.3 (± 0.2)	3.6 (± 3.1)	1.0 (± 0.6)	0.5 (± 0.3)	1.8 (± 1.1)
Zn	10.3 (± 4.7)	4.0 (± 1.8)	3.6 (± 2.6)	11.6 (± 6.1)	4.5 (± 1.7)	3.6 (± 2.7)	1.2 (± 0.6)

n.d. : not determined as the concentration in the parasite was below or around the detection limit.

**Table 4:** Spearman correlation coefficients (r) for the significant relationships between element concentrations in parasites and fish tissues

**Element**	**Parasite versus organ/parasite**	**R**	**p**
Co	*Eustrongylides *sp. ↔ intestine	0.53	< 0.05
	*Eustrongylides *sp. ↔ liver	0.51	< 0.05
	*Eustrongylides *sp. ↔ *P. laevis*	0.53	< 0.05
	*P. laevis *↔ intestine	0.78	< 0.01
	*P. laevis *↔ liver	0.74	< 0.01
Cd	*Eustrongylides *sp. ↔ muscle	0.79	< 0.01
	*Eustrongylides *sp. ↔ liver	0.66	< 0.05
Fe	*Eustrongylides *sp. ↔ muscle	0.57	< 0.05
	*Eustrongylides *sp. ↔ liver	0.62	< 0.05
Mn	*Eustrongylides *sp. ↔ intestine	0.69	< 0.01
	*Eustrongylides *sp. ↔ *P. laevis*	0.69	< 0.01
	*P. laevis *↔ intestine	0.53	< 0.05
	*P. laevis *↔ liver	0.71	< 0.01
Pb	*Eustrongylides *sp. ↔ muscle	0.53	< 0.05
	*Eustrongylides *sp. ↔ liver	0.74	< 0.01
	*Eustrongylides *sp. ↔ *P. laevis*	0.68	< 0.01
	*P. laevis *↔ liver	0.55	< 0.05
Se	*Eustrongylides *sp. ↔ muscle	−0.69	< 0.01
	*P. laevis *↔ muscle	−0.51	< 0.05
Sn	*Eustrongylides *sp. ↔ muscle	−0.72	< 0.01
V	*Eustrongylides *sp. ↔ intestine	0.68	< 0.01
	*Eustrongylides *sp. ↔ liver	0.56	< 0.05
	*Eustrongylides *sp. ↔ *P. laevis*	0.57	< 0.05
	*P. laevis *↔ intestine	0.69	< 0.01

Comparisons of element concentrations between larval *Eustrongylides* sp. and fish tissues showed that almost all essential elements (Co, Cu, Fe, Se and Zn) were significantly higher accumulated in the nematodes (Wilcoxon test, p < 0.01; see Table 2). According to the bioconcentration factors the nematodes showed the highest accumulation rates for Cu and Co followed by, Fe, Se and Zn (Table 3). The accumulation of toxic elements, however, showed some differences. The concentrations of Pb in *Eustrongylides* sp. were significantly higher (Wicoxon test, p ≤ 0.01) compared to muscle and liver, but its concentrations remained similar to those in the intestine. The levels of Cd in the nematodes were significantly lower or similar to those in the host tissues in contrast to Sn, which was accumulated to a higher degree in the parasite (Wicoxon test, p < 0.05; Table 2). The mean BCFs for the latter were 55, 22 and 9 for muscle, intestine and liver, respectively (Table 3). Correlation analyses between element concentrations in nematodes and fish organs showed more significant associations in comparison to acanthocephalans. The levels of eight metals (Co, Cd, Fe, Mn, Pb, Se, Sn and V) in organs correlated with those in *Eustrongylides* sp., from which most of them (Cd, Fe, Pb, Se, Sn) showed significant associations with the concentrations in muscle. The levels of toxic elements like Cd and Pb in larval nematodes were positively correlated with those in muscle and liver (see Table 4). The only negative associations were obtained for the elements Se (r = − 0.69; p < 0.01) and Sn (r = − 0.72; p < 0.01) between concentrations in the nematode and muscle tissue.

The comparisons of element concentrations between both parasite taxa revealed clear differences. Essential elements like Co, Cu, Fe, Se, Sn and V were found in significantly higher concentrations in the larval *Eustrongylides* sp., whereas the adult *P. laevis* accumulated the elements As, Cd, Mn and Pb to a significantly higher degree (Table 2 and Table 3, Figure 1). Accordingly, the ratios (C_[*Eustrongylides* sp.]_ / C_[*P. laevis*]_) for most essential elements were higher than 1, with the following values in decreasing order: Cu > Co > Fe > Se > V > Zn. In contrast, the concentrations for As, Cd, Mn and Pb, were between 1.7 to 25 times higher in *P. laevis* (Table 3) compared to *Eustrongylides* sp. Correlation analyses between element concentrations in acanthocephalans and nematodes revealed significant associations only for the elements Co, Mn, Pb and V, (Table 4).

## Discussion

Our field metal monitoring compares and highlights the accumulation potential of different fish helminths. Nine of eleven elements were found in significantly higher concentrations in the parasites in comparison to the host tissues. However, the range of elements in parasites differed between parasite taxa. The acanthocephalans accumulated primarily elements which are known for their toxicity to many organisms such as As, Cd and Pb [19]. In contrast, the larval nematodes mainly bioconcentrated elements which are part of several enzymes and other macro-molecules and which are therefore considered as essential elements (Co, Cu, Fe, Se, Zn) for many organisms [19]. Different authors reported high accumulation rates of heavy metals in acanthocephalans and demonstrated that they can be successfully used for monitoring purposes (summarized by [2,20]). As expected, our study confirmed these results for the acanthocephalan *P. laevi*s, which is probably the most intensively investigated fish acanthocephalan regarding metal accumulation [4]. Our data corresponded to field data published from the Danube River [17,21,22] and showed some parallels with the metal uptake experiments performed under controlled laboratory conditions [7,13,23]. Again, concentrations of As, Cd, Cu, Mn, Pb and Zn in *P. laevis* exceeded the levels in the fish host, which confirms the use of acanthocephalans as indicators for metal pollution.

Metal monitoring studies performed with the help of parasitic nematodes are comparatively scarce. Most of the available nematode papers focus on the accumulation potential of adult parasites, while larval nematodes are less intensively investigated and information about their accumulation capacity is missing. Our study focused on fourth stage larvae, which were able to accumulate a large number of elements, especially essential ones (Co, Cu, Fe, Se, Zn). This demonstrates that metal uptake already starts during an early stage of development. In contrast, the accumulation capacity of larval acanthocephalans (cystacanths) was found to be very low and the metal uptake starts in the intestinal lumen of their definitive host [24-26].

The higher levels of essential elements in the nematodes could be related to their biology and morphology. After the fish acquires the infection, the larva migrates through the intestinal wall into the body cavity, where it starts feeding on blood and tissues prior to encapsulation. Similar to adult nematodes, the fourth stage larvae have a completely developed digestive system [27], which suggests that they can accumulate metals by ingestion of food. Research on microstructure and properties of the nematode’s cuticle revealed that the cuticle of larvae is not as complex as that of adults [28,29]. Therefore, larval nematodes are also able to adsorb nutrients and metals through their body surface. Taken together it appears that fourth stage nematodes exhibit an even better accumulation capacity than adult stages because of different uptake routes. This was probably one of the reasons why essential metals like Cu, Fe, Zn as well as Co and Se were predominantly accumulated. These macro and micro elements are important structural and functional factors, as they are involved in the architecture of many enzymes and other complex molecules [19]. For example, elements like Fe were adsorbed or ingested most likely with host blood, as Fe is an essential part of the blood pigment hemoglobin. Therefore, its levels in host muscle and liver tissues correlated with the levels in the nematodes, (see Table 4). Similar uptake sources may also exist for the elements Co, Cu and Zn due to the fact that these elements are highly abundant in organisms as co-factors of various enzymes. Correlation analyses between Co levels in nematodes and host liver or intestine for example revealed positive associations, which again underlines that the parasites profit from high levels in the host and were not able to negatively affect the balance of microelements in their host.

Higher metal concentrations in the larval lung nematode *Pseudalius inflexus* were reported [29] not only for essential elements but also for toxic ones. The authors suggested that L4 larvae accumulated metals mainly from the food (blood and host tissues) via their digestive system. However, this nematode was not encapsulated, as was the case for *Eustrongylides* sp., therefore element uptake will not only occur via food, but also via the cuticle. This assumption is supported by the fact that levels of toxic elements such as As, Cd and Pb in the parasite were similar to those in the host tissues. More specifically, the concentrations of Cd and Pb in nematodes were positively correlated with those in muscle and liver, indicating that these metals were taken up from the tissues in which the parasites were located (for details see Table 4). Obviously, the specific microhabitat preferences of *Eustrongylides* sp. play a decisive role, as the availability of toxic elements within the fish host differs profoundly from those of the essential ones. With reference to this, high levels of Cd and Pb in larval nematodes of the genus *Hysterothylacium* sp. collected from the intestinal lumen and from mesenteries of the fish host have been reported recently [30]. It seems that both metals were available to a high degree in the digestive tract which therefore results in metal accumulation rates similar to acanthocephalans.

Higher concentrations of various elements were also found in the adult nematodes *Philometra ciprinirutili* and *P. ovata* inhabiting the body cavity of fish [10,12]. Interestingly, the authors reported higher levels of non-essential elements like Cd and Pb in the parasites in contrast to the results obtained in the present study. Mean ratios of Cd and Pb between *P. ovata* and host muscle ranged between 20 and 25 [12], which indicates a much higher accumulation capacity of Philometrids in comparison to *Eustrongylides* sp. On the other hand, the respective bioaccumulation rates for Cu (123) and Zn (12) in our study were much higher than those reported for *P. ovata*[12] with only 22 and 3, respectively. These differences suggest that larval nematodes probably have a higher affinity to accumulate essential elements whereas the adult Philometrids demonstrated a higher accumulation capacity for toxic metals. Explanations for these differences could be the relative importance of different element uptake routes between the developmental stages of the nematodes or competition between *Eustrongylides* sp. and *P. laevis* in the double infected fish. The nematode larvae were encapsulated and thus were unable to feed actively in contrast to the adult stage. As the larval stages have to grow fast during their development they rely on the uptake of essential elements probably via uptake processes through their cuticle. Adult nematodes actively feed on host tissues and thereby take up and accumulate toxic metals like Cd and Pb.

An alternative explanation for the relatively low levels of toxic elements in *Eustrongylides* sp. could be competition between acanthocephalans and nematodes. It is suggested that acanthocephalans dwelling in the intestine compete for nutrients and metals with the host tissues [31] probably via interruption of the enterohepatic element cycle [7]. Metals bound in bile complexes excreted in the small intestine are taken up by acanthocephalans and thus are not available for reabsorption by the host intestine. Therefore, toxic elements might become unavailable for the host target tissues and simultaneously occurring in parasites. In our study, the concentrations of Pb in *Eustrongylides* sp. were significantly higher in comparison to the muscle and liver tissues, but not higher than those in the intestine, which supports the assumption, that some heavy metals are predominantly available for intestinal parasites. In mass infection cases with *P. laevis*, which are common for barbel [15,32], metal distribution in the host tissues might be significantly changed by the acanthocephalans.

## Conclusions

Due to the taxa specific localization in the host and differences in the development stage, the nematodes and acanthocephalans exhibited different accumulation profiles. Larval *Eustrongylides* sp. accumulated mainly essential elements, whereas the adult *P. laevis* showed a higher affinity to take up non-essential (toxic) elements. Our results suggest that larval nematodes can also be applied as sensitive indicators for metal pollution. It could be even advantageous if they are taken as sentinels in addition to acanthocephalans due to their complementary accumulation profile. Using this host-parasite system a large number of elements could be analyzed, therefore it represents an appropriate tool for future metal monitoring surveys if low environmental levels have to be detected.

## Abbreviations

BCF: Bioconcentration factors.

## Competing interests

The authors declare that they have no competing interests.

## Authors’ contributions

MN was involved in sampling, parasitological investigations, metal analyses and in the data processing and evaluation as well as in writing of the manuscript. GS contributed to the analytical work (element analysis) and data processing. BS played a substantial role in the writing process by corrections and critical comments and in the conception and guidance of the study. All authors read and approved the final version of the manuscript.
